# Drug utilization patterns and reported health status in ethnic German migrants (Aussiedler) in Germany: a cross-sectional study

**DOI:** 10.1186/1471-2458-11-509

**Published:** 2011-06-28

**Authors:** Anna Volodina, Thilo Bertsche, Karel Kostev, Volker Winkler, Walter Emil Haefeli, Heiko Becher

**Affiliations:** 1 Institute of Public Health, University of Heidelberg, Germany; 2 Department of Clinical Pharmacology and Pharmacoepidemiology, University of Heidelberg, Germany; 3 Co-operation: Unit Clinical Pharmacy, University of Heidelberg, Germany; 4 IMS HEALTH GmbH & Co. OHG, Centre of Excellence Patient Data, Frankfurt am Main, Germany; 5 Institute of Pharmacy, Department of Clinical Pharmacy, Eilenburger Str. 15a, 04317 Leipzig, Germany

## Abstract

**Background:**

Inadequate utilization of healthcare services by migrant populations is an important public health concern. Inadequate drug consumption and poor compliance to the therapeutic regimen are common manifestations of low health-care seeking behavior present in migrants even in the countries with well-established healthcare systems. There are few studies on the use of medicines among the different groups of migrants in Germany. The objective of this study is to investigate drug consumption patterns of ethnic German migrants (Aussiedler) and their current health status.

**Methods:**

A cross-sectional study nested into a cohort of 18,621 individuals aged 20-70 years who migrated to Germany from the former Soviet Union between 1990 and 2005 was conducted. Data on consumption of drugs, drug handling, major health risk factors, and one-year disease prevalence were obtained for 114 individuals through a self-administered questionnaire and phone interviews. Results were compared to the data on the German population derived from the Disease Analyzer database and Robert Koch Institute (RKI) annual reports. Direct age standardization, test of differences, Chi-square test, and descriptive statistics were applied as appropriate. For drug classification the Anatomical Therapeutic Chemical (ATC) system was used.

**Results:**

Of the respondents, 97% reported to have at least one disease within a 12-month period. The one-year prevalence of asthma (6.9%), hypertension (26.7%), chronic bronchitis (8.6%), and diabetes (4.9%) in migrants was similar to the general German population. 51% regularly took either over-the-counter (OTC) medication or prescription medicines. Six ATC groups were analyzed. The highest drug consumption was reported for the ATC cardiovascular (22%), nervous (9%), and muskulo-skeletal system (8%). 30% used OTC medicines obtained in the country of origin. Difficulties with drug handling were rare. Alcohol consumption did not differ from the German population (p = 0.19 males and 0.27 females), however smoking prevalence was lower (p < 0.01) in both sexes.

**Conclusion:**

Ethnic German migrants seem to differ only slightly from Germans in health status, drug utilization, and disease risk factors, and if so, not in an extreme way. Country of origin remains a source of medicines for a substantial part of migrants. The study is limited by a small sample size and low response rate.

## Background

In light of increased global migration, integration of migrant populations into the healthcare system of a receiving country becomes a key public health concern. Even in western industrialized countries where access to health services in the general population is ensured, health care utilization rates are usually lower for migrants compared to the autochthonous population [1-3]. Many studies in western countries have identified a common health care utilization pattern of migrants characterized by fewer visits to specialists or primary care units and less utilization of preventive services, such as cancer screening or child health services [4,5]. Consequently, migrants tend to visit emergency rooms more frequent than the general population [6-8]. Health-care seeking behavior of the migrants is influenced by a number of socioeconomic, cultural, and individual factors, such as linguistic barriers, lack of knowledge about available medical services, different perception of health, disease, and sickness, disapproving attitude to the health services and personnel in the host country, personal health practices, and many more.

By addressing this issue in the "Health of Migrants" report of 2007, the World Health Organization (WHO) highlights the vulnerability of migrants regardless of type and reason of migration and emphases on eliminating the differences in health status between migrants and the native population. It demands that every national healthcare system has advanced mechanisms in place to address migrants' health needs and to provide migrant sensitive interventions [9].

Use of medicines constitutes an important part of many medical treatments and disease prevention interventions. Some studies on drug utilization in Spain have observed that immigrants consume fewer medicines and have much lower expenditures on pharmaceuticals compared to the autochthonous population [10,11].

Another aspect modulating drug utilization patterns of migrants is the possible lack of trust in the proposed treatment and, as a consequence, poor compliance. Several pharmaco-epidemiological studies on different migrant populations reported the use of self-management practices, traditional remedies, or self-imported medicines before or instead of seeking help within the hosting healthcare system [12-15]. A study on Pakistan migrants in England reported a preference for traditional medicines to conventional Western medicines and highlighted the need to consider possible toxicity and interactions between them if used concurrently [14]. Another study on Turkish immigrants in Germany discovered a wide use of plant-based remedies that were not in use in German Phytotherapy [15]. Such practices may oppose the effects of conventional treatment and are difficult to monitor.

Ethnic German migrants (in German called "Aussiedler") from the former Soviet Union are a unique group of diaspora migrants. Since 1990, more than two million moved to Germany. Today, they constitute the second biggest group of migrants living in Germany and represent about 2.5% of the German population [16]. Although they are German citizens by law, many of them face similar problems as other immigrant groups [17].

Information on the current health status of migrants in the scientific literature is controversial. A representative study on comparison of ethnic German migrants with the German population, conducted by the Cooperative Health Research in the Region of Augsburg in 2000, has identified them as a high risk population group, due to the higher prevalence of risk factors such as overweight, high cholesterol, and poor use of preventive services [18]. In contrast, several studies performed at the University of Heidelberg demonstrated that migrants have significantly lower overall mortality than the overall German population [19-21]. Scientific knowledge on health related issues in this migrant group, such as prevalence of common risk factors, drug consumption patterns, and utilization of drugs is sparse.

The correct use of drugs is a major issue in pharmacotherapy. Medication errors are frequent in a hospital setting and often caused by inappropriate drug handling and administration [22]. Systematic strategies for their prevention, however, are still lacking. A previous study investigated the prevalence and potential risk of medication handling errors, corresponding knowledge deficits in nurses and parents committing the errors [23]. In over 40% (among nurses) and 96% (among parents) of all observed processes medication handling and administration errors were detected [23]. We postulate that this proportion would be higher in the migrant population, due to language barriers, and different perception and cultural background may add to the percentage.

Here, we report results of a cross-sectional study nested into a cohort of migrants from the former Soviet Union on drug consumption, handling and administering drugs in the light of reported health status and socio-demographic characteristics of this population.

## Methods

This is a cross-sectional study nested into a large cohort of 18,621 migrants, who have migrated to Saarland, a federal state of Germany, from the former Soviet Union between 1990 and 2005 [21]. Personal information on migrants was obtained from all 7 local refugee offices and included the following items: name, sex, date and country of birth, date of migration, vital status, and initial address. 2576 members of this cohort with current age between 20 and 70 years and whose home addresses were available, formed the initial sample for this study. The study was approved by the responsible Ethics Committee of the Medical Faculty of Heidelberg University.

A study population of 30 percent (792 individuals) was randomly selected from the initial sample. A structured questionnaire with twenty-two questions on demographic data, current health status, major risk factors (smoking, alcohol consumption), current diseases and former severe diseases, and current drug intake was developed in German and Russian to provide responding participants a choice of the language. Pre-testing of the questionnaire and subsequent refinement was done with nine migrants.

Current professions of the respondents were classified into the major groups according to the 2000 Standard Occupational Classification system developed by the United States Bureau of Labor Statistics. To assess major groups of medicines utilized by migrants the participants were asked to specify the name and pharmaceutical form of the medicines that they were currently taking. Reported medicines were classified according to the ATC Classification system, which divides drugs according to their therapeutic and chemical characteristics and is widely used as an international tool in drug utilization studies [24]. Drug consumption was assessed by counting the number of prescriptions from each of the ATC groups.

Data collection took place from March to September 2009. In a first period (March to May) the self-administered questionnaire was sent by mail to all 792 individuals. 82 letters were returned due to invalid addresses resulting in a trial cohort of 710 persons. After two weeks one reminder was sent to the non-respondents. In September 2009, 257 of 559 non-responders, whose phone numbers were listed in the phone book, were contacted by phone. One follow-up call was done in case a person was not able to participate in the survey during the first contact. Overall 200 interviews were conducted by phone either in Russian or German by one of the authors (A.V.). Data from 114 individuals were obtained (41% with phone interview), which corresponds to a response rate of 23.5% among those contacted by phone and 16% (114/710) overall.

For comparison with the general population living in Germany, data from the RKI and Disease Analyzer [25] were used. Disease prevalence estimates were obtained from the RKI health survey of 2002/2003, a representative sample (n = 8,318) of the German population over 18 years [26].

Information on drug intake in the general population in Germany was obtained from a representative sample (n = 1,417,290) in the age range 20-70 years, who had at least one visit of a general practitioner (GP) from June 2009 to August 2009. This sample has been derived from the Disease Analyzer, a large patient database that allows anonymous access to 964 general and internal medicine practices all over Germany [27]. The data are generated directly from the computer in the physician's practice via standardized interfaces and provide daily routine information on diseases and therapies of more than 20 million patients in Germany. Derived information is checked for plausibility, linked with relevant additional information such as price of a medicinal product, saved, and updated on a monthly basis. The Disease Analyzer includes only anonymized data in compliance with the requirements of the German data protection laws.

An estimate of the proportion of the population that has at least one visit per year at a GP in Germany was obtained from the drug consumption report of the Gmünder Ersatzkasse (GEK), a large health insurer in Germany.

### Statistical methods

To compare prevalences we performed a direct age standardization of the study population to the age distribution in Germany, taken from the WHO Statistical Information System [28]. The RKI sample was representative for the age range. Tests of differences were performed for comparison of disease prevalence, drug consumption, smoking, and alcohol consumption among migrants and the German population using a Chi-square test.

## Results

### Epidemiological and socio-economic characteristics

114 migrants from the four former Soviet Union countries Russia, Ukraine, Georgia, and Kazakhstan participated in the survey. Their mean age at the time of migration was 36 years. Peak immigration was between 1994 and 1996.

The sample included 50% males (57/114), mean age of the sample was 49.7 ± 13 years, range 21 to 70 (males 50.2 ± 13 range 21 to 69, females 49.7 ± 12 years, range 23 to 70) (Table 1). Socioeconomic status was assessed by education, current occupation, and current employment status. Most respondents (68%, 63% in males, and 73% in females) had tertiary education and 31% had completed primary or secondary school. Female respondents were mainly occupied in educational and healthcare support sectors and worked as teachers or paramedical personnel (nurse, laboratory assistant). Overall, 55% were professionally active (full or part time). Among those, 85% were semi-skilled workers. The language mainly spoken at home was assessed as an indirect indicator of integration level into the new socio-cultural environment. 87% of the respondents continued using Russian language at home either completely or partly. During the mail and phone survey, 39 of 114 questionnaires were completed in German and 75 (66%) in Russian.

**Table 1:** Demographic characteristics of the sample under study

		N	Percent
**Sex**	Males	57	50
	Females	57	50

**Age (years)**	20-29	11	9.6
	30-39	16	14
	40-49	23	20.2
	50-59	35	30.7
	60-79	29	25.4
			

**Education**	Higher education	41	35.9
	Technical college	36	31.6
	Secondary education	24	21.1
	Primary education	11	9.6
	No education	2	1.8
			

**Employment status**	Student	4	3.6
	Unemployed	17	14.9
	Part-time employment	19	16.6
	Full-time employment	47	41.2
	Retired	27	23.6

### Current health status and common risk factors

Main reported health problems within the last 12 months were back pain (66 cases), hypertension (36), and insomnia (27). Other prevalent health conditions were kidney disorders (11), chronic bronchitis (9), diabetes (8), and asthma (7). 97% of the respondents reported at least one health problem during the last year. The age-standardized disease frequencies were similar to the German population with the exception of back pain which was significantly more frequent in migrants (55% compared to 18.6%; p < 0.001, Table 2). The proportion of the population which had at least one GP visit per year was estimated as 72% for the age range 20 to 70 years [29].

**Table 2:** Age standardized disease prevalence among migrants, compared to the data on the German population

Disease	One year period prevalence		p - value
	Migrants^1^	Germany (RKI)	
Asthma	7%	6%	0.61
Back pain	55%	19%	< 0.001
Diabetes	5%	6%	0.98
Chronic bronchitis	9%	8%	0.88
Hypertension	27%	27%	0.93

^1 ^age-standardized to German population

Self-assessment of the current health status was not significantly different from the German population and was independent of sex: most respondents indicated their health status as good, extreme assessments such as "very good" or "very bad" were rare (n = 17). However, the proportion of migrants who reported some degree of dissatisfaction was significantly higher when compared to the German population (25.5% and 11% respectively, p < 0.01) [30].

A comparison of smoking and alcohol consumption as two main health risk factors between the respondents and the overall population in Germany is given in Table 3. While alcohol consumption appears to be similar, there was a significantly lower prevalence of smoking in migrants for both sexes (p < 0.01). This comparison is for both populations at large, and not adjusted for socioeconomic levels.

**Table 3:** Smoking and alcohol consumption among migrants and the German population

			Migrants	German population	p-value
Current alcohol consumption	Males	No	19%	12%	0.19
		Seldom/special occasions	46%	52%	
		Moderate to heavy	35%	36%	

	Females	No	23%	24%	0.27
		Seldom/special occasions	68%	60%	
		Moderate to heavy	9%	16%	

Smoking	Males	Never smoked	49%	33%	< 0.01
		Former smoker	12%	31%	
		Current smoker	39%	37%	

	Females	Never smoked	79%	52%	< 0.01
		Former smoker	5%	22%	
		Current smoker	16%	27%	

^1 ^age-standardized to German population

### Drug consumption and sources of drug purchase

**Figure 1:**
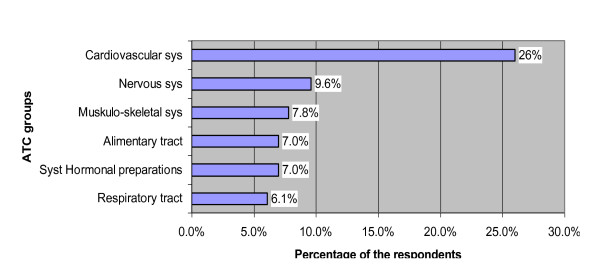
**ATC groups of medicines consumed by the respondents**. Main ATC groups of medicines consumed by the respondents (%) within the 12-months period.

51% of the respondents reported regularly taking either OTC or prescription drugs; the main ATC groups of consumed medicines are cardiovascular, nervous, and muskulo-skeletal system drugs (Figure 1). Overall 30% of the respondents continued using one or several medicines from the country of origin.

Migrants' drug consumption was compared to the sample derived from the Disease Analyzer. Both samples were age standardized to the German population using direct standardization (Table 4). For identified ATC groups differences in consumption rates were not significant except for cardiovascular drugs, where migrants consumed twice as many medicines as the German population (p = 0.005).

**Table 4:** Age-standardized drug consumption of the respondents compared to the German population

ATC code	Age-standardized^1 ^drug consumption		p-value Test of difference
	Migrants	Germany (Disease Analyzer)	
A. Alimentary tract and metabolism	6.6%	8%	0.55
C. Cardiovascular system	22%	11%	0.005
H. Systemic hormonal preparations, excl. sex hormones and insulins	6%	5%	0.56
M. Musculo-skeletal System	8%	6%	0.37
N. Nervous system	9%	6%	0.27
R. Respiratory system	6%	4%	0.30

^1 ^age-standardized to German population

### Handling of medicines

To assess possible problems with drug handling, participants were asked whether they experience any difficulties in using their medicines, and if yes, to specify these difficulties. The majority of respondents (79%) stated that they did not have any difficulties in handling their drugs. 68% of the respondents stated also that they go to a Russian-speaking GP to avoid language and cultural barriers. Numbers are too low for a more detailed analysis.

## Discussion and conclusion

This study attempted to investigate aspects of the current health status, main health risk factors, and drug consumption patterns of ethnic German migrants living in Germany in comparison to the native German population. Despite some major limitations as discussed below, the main results appear noteworthy:

Age-standardized prevalence of hypertension, chronic bronchitis, diabetes, and asthma in migrants is similar to the German population, except prevalence of back pain. Here, it must be noted that the RKI definition of back pain is based on physician diagnosis whereas here it is based on self-report. Studies on self-reported back pain in Germany give much higher prevalence rates [31]. A representative nationwide study conducted by the National German Health Survey in 2005 reported one year prevalence rate of 60% [32] which is more similar to the prevalence found in our migrant sample.

Smoking and alcohol consumption are main risk factors for several chronic diseases. Both Germany and Russia have relatively high per capita alcohol consumption: 12.0 liters of pure alcohol in Germany and 10.3 liters of pure alcohol in Russia [33]. Empirical studies on alcohol consumption patterns of migrants have found no significant differences in the consumption patterns between migrants and the German population [34,35]. With respect to smoking, several studies on migrants have observed higher prevalence of smokers among males and youth and lower among females, when compared to the overall population [36-38]. Again, this is consistent with the results of this study.

The information on drug consumption patterns of migrants in Germany, in particular Aussiedler, is scarce in the literature. Most of the studies refer to the consumption of illegal drugs and psychoactive substances by young migrants and associated violence [38,39]. Several epidemiological studies on the consumption of medicaments among migrants in other countries observed lower consumption rates compared to the general population and more use of low-priced medicines, concluding that traditional beliefs should be taken into account by the health care services when developing health policies targeting a migrant population [5,8,14].

51% of the respondents regularly take either OTC or prescription drugs. Most medicines belong to three ATC groups for the treatment of cardiovascular, nervous, and muskulo-skeletal system. These groups correspond to the three most common health problems stated by the respondents.

In agreement with our initial hypothesis, many (30%) of the respondents purchased drugs from their home country either during their visits or through relatives. Drugs obtained abroad belong to the OTC group and represent mainly topical products for joint and muscular pain and analgesics. There was no evidence that this practice is harmful to migrants. 21% of the respondents indicated that they experience difficulties with handling of drugs obtained in Germany, related to the understanding of patient information leaflets and complying with the therapy regimen. 68% prefer to visit Russian-speaking physician when seeking medical help, who clearly explains the proper handling of medicines and dosage regimen.

Socioeconomic characteristics of migrants were explored since they have an impact on the utilization of the health services and therefore on the consumption of medicines. Most respondents actively use Russian language within their close circle of family and friends. Educational level of the migrants was higher when compared to the German population: 68% completed tertiary education, whereas in Germany this corresponds to 24%. However differences in the educational system between the former Soviet Union countries and Germany must be taken into consideration.

The study has a number of limitations: first, the response rate was too low to allow a generalization of the results on the overall population of ethnic German migrants in Germany. The distribution of the occupation in females suggests that a bias may have occurred towards a better health seeking behavior, fewer problems with drug handling, and a lower proportion of risk factor exposure. We acknowledge that this may introduce selection bias in the study. High response rates, however, are particularly difficult to achieve in studies on migrants, and alternative studies, like qualitative studies and focus group discussions would add useful information here.

Second, the information on the use of medicines is based on the data from the GEK report, which includes information only on the GEK insured population. However, this is the only published large source of data on drug consumption available. Third, results on health risk factors obtained from self-reporting must be interpreted carefully since certain types of response bias may be introduced. The respondents may have a tendency to give socially desirable answers or only migrants following a healthy life style may have participated in the survey. Lastly, the overall sample size was small and precludes definite conclusions even if all other limitations had not occurred.

It is generally difficult to motivate migrant groups to participate in surveys, and this study must be seen in the light of this fact. Overall, we conclude that ethnic German migrants seem to differ from Germans in only a few aspects related to health status, drug utilization, and disease risk factors, and if so, not in an extreme way. This is consistent with the observed mortality pattern from the original large cohort study on which this survey was based [19,20] where we observed some differences in the mortality pattern, however in a moderate magnitude. Nevertheless, larger studies on consumption of medicines among different groups of migrants should be performed. It would be interesting to identify groups of drugs within the ATC system where the consumption is different from the overall population and the causes of these differences. Also, questions on the use of traditional remedies and adherence to therapy should be addressed further.

## List of abbreviations

ATC: Anatomic Therapeutic Chemical; GEK: Gmünder Ersatzkasse; GP: General Practitioner; OTC: Over-the-Counter; RKI: Robert Koch Institute; WHO: World Health Organization.

## Competing interests

The authors declare that they have no competing interests.

## Authors' contributions

AV participated in the design of the study and acquisition of the data, performed descriptive analyses, and drafted the manuscript. HB conceived the study, participated in its design and its coordination, performed the statistical analyses, and contributed to the writing of the paper. TB and WEH participated in the design of the study, development of the questionnaire, and contributed to the writing of the paper. VW participated in the study design and development of the database. KK helped to analyze the data and draft the manuscript. All authors read and approved the final manuscript.

## Pre-publication history

The pre-publication history for this paper can be accessed here:

http://www.biomedcentral.com/1471-2458/11/509/prepub
